# Strengthening the bond with the scientific community: *FEBS Open Bio* in 2025

**DOI:** 10.1002/2211-5463.13956

**Published:** 2025-01-06

**Authors:** Sara Fuentes, Miguel A. De la Rosa

**Affiliations:** ^1^ FEBS Open Bio Editorial Office Cambridge UK; ^2^ Institute for Chemical Research (IIQ), Scientific Research Centre Isla de la Cartuja (cicCartuja), Universidad de Sevilla‐CSIC Sevilla Spain

## Abstract

*FEBS Open Bio* remains dedicated to serving the scientific community by ensuring rapid publication of rigorous science and pioneering initiatives to support researchers. In this editorial, we reflect on a year of achievements, and look forward to the new developments planned for 2025.

*FEBS Open Bio* is one of the four not‐for‐profit journals owned by the Federation of European Biochemical Societies (FEBS). A key difference in the editorial policy of *FEBS Open Bio* compared with the other three FEBS journals is its focus on the soundness of science rather than on the impact and perceived significance of the research. Decisions are based on scientific validity, robust methodology and high ethical standards, and all submissions, including exploratory, confirmatory and descriptive work, are considered provided they make a meaningful contribution to the scientific community. The scope of the journal is broad and includes biochemistry, molecular and cell biology, bioinformatics, microbiology, virology, developmental biology, neurobiology, immunology and related disciplines. *FEBS Open Bio* also publishes articles on biochemistry education research.

## Publication ethics

As AI tools become integral to academic research and writing, journals face growing challenges to uphold high ethical standards. It is vital for journal staff to keep up to date not only with the new AI tools and their improved functionalities but also understand how these might be used by authors to ensure their usage is adequately disclosed. In 2024, *FEBS Open Bio* together with the rest of the FEBS Press journals, updated its AI disclosure policies to clearly define what is the permissible use of generative AI or AI‐assisted tools and how their use should be disclosed. Our full AI disclosure policies can be found here: https://febs.onlinelibrary.wiley.com/hub/author‐policies#AI

The use of AI can introduce errors and inaccuracies, and although authors remain responsible to review any AI‐generated output, FEBS Press journals have proactively adopted measures to address these issues. Over the past year, we have implemented several new tools to help combat errors as well as misconduct and intentional deceiving practices. The rapid development of these tools together with the work of our in‐house image integrity analyst, Jana Christopher, put *FEBS Open Bio* and the rest of FEBS Press journals in a strong position to meet future challenges.

## Open access: Open data and preprints

The open access movement, which began in the early 1990s, has consistently removed barriers to make research outputs as open and accessible as possible. We are proud to announce that as part of our efforts to advance open access practices, *FEBS Open Bio*, along with the rest of FEBS Press journals, partnered this year with Dryad to support open data sharing. By depositing their data to Dryad, FEBS Press authors can contribute to accelerating the rate of discovery and help combat the reproducibility crisis.

We are also excited to share that FEBS Press journals have joined Review Commons, a platform that enables high‐quality journal‐independent peer review. Authors submitting to Review Commons are provided with a Refereed Preprint, which allows their manuscript to be considered by any of the 28 affiliated journals, including *FEBS Open Bio*.

## Supporting young scientists

Since its launch in late 2011, *FEBS Open Bio* has been committed to the rapid publication of sound science and the support of the wider scientific community. In 2024, we were proud to award over 35 *FEBS Open Bio* Poster and Oral presentation prizes to early‐career researchers at 30 international meetings and conferences, including two speed talk prizes and a poster prize at the 48th FEBS Congress.

Last year, we also awarded our annual *FEBS Open Bio* Article Prize to an early‐career researcher who had authored a paper of special interest published in the journal in the preceding year. The 2024 prize was awarded to Klaudia Jączyńska (UT Southwestern Medical Center, Dallas, TX, USA) as the first author of the outstanding paper ‘Analysis of tripartite Synaptotagmin‐1‐SNARE‐complexin‐1 complexes in solution’ [1]. The winning paper was selected by a jury comprised of three members of the journal's Editorial Board: Stuart Ferguson (Oxford), Takashi Gojobori (Mishima) and Dietmar Manstein (Hannover). As part of the prize, Klaudia received a travel and accommodation bursary to attend the 48th FEBS Congress, where she delivered an excellent presentation.

We are delighted to support and recognise the work of so many early‐career researchers, and we would like to once again congratulate all *FEBS Open Bio* prize winners.

In addition to the direct contributions made by *FEBS Open Bio*, the revenue raised through article publication charges from all FEBS Press journals is used to support the charitable activities of FEBS, including Advanced Courses, fellowships and grants, awards, and the FEBS congress.

## Content highlights: ‘In the Limelight’ special issues

In 2024, *FEBS Open Bio* continued its tradition of publishing its ‘In the Limelight’ Special Issues, which focus on timely topics through curated Review articles. This year, we published three Special Issues focussed on Alzheimer's disease (AD), toxicology and mitochondrial biogenesis.

Koji Yamanaka, Professor at the Nagoya University and *FEBS Open Bio* Editorial Board member, served as a guest editor for our special AD issue. This issue comprised four Review articles focussed on targeting and phosphorylation of tau, brain lipid metabolism and microglia heterogeneity. In the first article, Sahara and Higuchi [2] gave a comprehensive overview of recent advances in tau biology, *in vivo* diagnostic imaging and the development of tau‐targeted therapies. In the second Review, Kimura *et al*. [3] summarised the structural diversity of tauopathy and its implication in disease and biomarkers. In the third article, Kawade and Yamanaka [4] focussed on brain lipid metabolism in AD and its implication in oligodendrocyte abnormalities, an often‐overlooked aspect of AD. In the final article, Dadwal and Heneka [5] focussed on microglia, innate immune cells in the brain, and their heterogeneity in neurodegenerative diseases.

Our second special issue of the year was guest‐edited by Frank Michelangeli, Emeritus Professor at the University of Chester, Honorary Professor at the University of Birmingham and *FEBS Open Bio* Editorial Board member. This special ‘In the Limelight’ issue highlighted the current and future research directions of environmental toxicology. In the first of the Review articles, Michelangeli *et al*. [6] focussed on the effects of endocrine disrupting chemicals, such as alkylphenols and brominated flame retardants, on Ca^2+^ transporters. In the second article, Erradhouani *et al*. [7] examined the effect of metabolic disrupting organic pollutants with particular emphasis on the intestine and highlighted how zebrafish might be a promising model system for the study of these chemicals. In the third and final Review, Eze and Vinken [8] addressed the toxicity of e‐waste at cellular and molecular levels and proposed some safety testing approaches.

Our final ‘In the Limelight’ issue of 2024 featured four Review articles and one Research article on mitochondrial biogenesis and was guest‐edited by Johannes A. Herrmann, Professor at the University of Kaiserslautern. The first Review article by Ganesan *et al*. [9] offered a comprehensive overview of the structure and function of mitochondrial β‐barrel outer membrane proteins. In the second article, Zarges and Riemer [10] focussed on the mitochondrial disulfide relay and the human pathologies associated with defects in this machinery. In the third Review article, Kizmaz *et al*. [11] examined the different import routes of proteins into the mitochondrial inner membrane. The last of the Review articles details factors involved in the mitochondrial ribosome assembly, with particular emphasis on thiol‐based redox processes as reviewed by Brischigliaro and colleagues [12]. This special issue also included an original Research article by Maruszczak *et al*. [13], which explored the structural conservation of the TIM23 complex, a key player in the mitochondrial protein import machinery, by comparing human and yeast TIM23 complexes. This issue also included an in‐depth interview with Hannes, where he explained the source of his scientific interest in mitochondrial biology [14].

To accompany the ‘In the Limelight’ issue on mitochondrial biogenesis, a free *FEBS Open Bio* webinar was held on November 19. Johannes A. Herrmann and contributing authors discussed key topics in mitochondrial research, with special emphasis on mitochondrial protein biogenesis. By exploring the molecular mechanisms underlying the different biogenesis routes of mitochondrial proteins, they highlighted the crucial role of mitochondria beyond energy metabolism and how we might come to think of mitochondria as the ‘beating heart of the cell’. The webinar was a great success with over 200 attendees from 32 different countries from Europe and overseas. The recording of the webinar, along with all our previous webinars, is available and free at: https://febs.onlinelibrary.wiley.com/journal/22115463/webinar.

## Broadening our boards

*FEBS Open Bio* covers a wide range of topics in biochemistry, biophysics, and cell & molecular biology as well as diverse article formats. To guarantee we continue to provide a fast, fair and thorough peer review to our authors in 2024, we have expanded our Editorial Board to welcome nine new scientists from across the globe:

- Aleksandra Mladenovic (Professor, Department of Neurobiology, Institute for Biological Research ‘Sinisa Stankovic’—National Institute of the Republic of Serbia, University of Belgrade, Belgrade, Serbia)
- Alberto Pascual (Professor, Instituto de Biomedicina de Sevilla (IBiS), Hospital Universitario Virgen del Rocio/CSIC/Universidad de Sevilla, Seville, Spain)
- Nesrin Kartal Özer (Professor, Uskudar University, Faculty of Medicine, Department of Biochemistry, 34 662, Istanbul, Türkiye)
- Heinz‐Peter Nasheuer (Professor, Centre for Chromosome Biology & Biochemistry, School of Biological and Chemical Sciences, University of Galway, Galway, Ireland)
- Ryuichi Morishita (Professor, Department of Clinical Gene Therapy, Center of Medical Innovation and Translational Research School of Medicine, Osaka University, Japan)
- Zrinka Kovarik (Associate Professor, University of Zagreb and Permanent Scientific Adviser, Institute for Medical Research and Occupational Health, Zagreb, Croatia)
- Deok Ryong Kim (Professor, Department of Biochemistry, College of Medicine, Gyeongsang National University, JinJu, South Korea)
- Graça Soveral (Professor, Department of Pharmaceutical Sciences and Medicines, Faculty of Pharmacy, University of Lisbon, Portugal)
- Paola Chiarugi (Professor, Faculty of Medicine and Surgery, University of Florence, Italy).

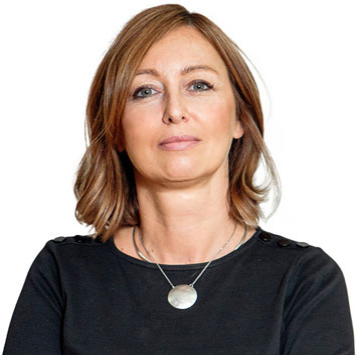

Aleksandra Mladenovic is a Research Professor at the University of Belgrade, Institute for Biological Research ‘Sinisa Stankovic’ (IBISS). She received her Ph.D. degree from the University of Belgrade, Serbia, and performed her postdoctoral training at the Laboratory for Molecular Neurobiology at IBISS and Laboratory for Molecular and Cellular Ageing at the National Hellenic Research Foundation, Greece. In 2003, she received a permanent position at IBISS. Her research focusses on the ageing brains, with particular emphasis on anti‐ageing dietary interventions, with the prime ambition to establish the optimal food intake pattern for healthy brain ageing.

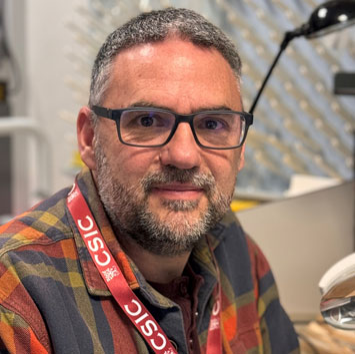

Alberto Pascual is a Principal Investigator at the Instituto de Biomedicina de Sevilla (IBiS, Spanish Research Council Investigator). He obtained his Ph.D. from the Universidad de Sevilla in 1999. He completed postdoctoral training at the Institute Alfred Fessard, CNRS, France and returned to Seville as a tenured scientist at the Hospital Universitario Virgen del Rocío. He started his own laboratory in 2011, and his recent research has been focussed on modifiable risk factors for Alzheimer's disease unravelling new pathogenic mechanisms.

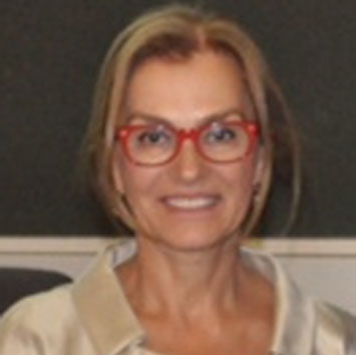

Nesrin Kartal Özer is a Full Professor at Uskudar University, Faculty of Medicine, Istanbul, Türkiye. Previously, she was a Visiting Scientist at St.George's Hospital, Medical School, London, UK; Institute of Biochemistry and Molecular Biology, University of Bern, Switzerland and University of Hohenheim, Stuttgart, Germany. Her research focusses on understanding how lipid metabolism impairs redox signalling and endoplasmic reticulum stress in metabolic and inflammatory diseases such as atherosclerosis and nonalcoholic fatty liver disease. In parallel, she has carried out research on the protective mechanisms of tocopherols and their metabolites in these diseases.

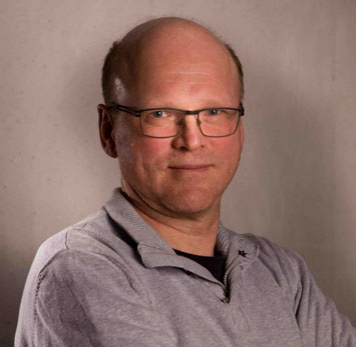

Heinz‐Peter Nasheuer is a Professor at the Centre for Chromosome Biology & Biochemistry in the School of Biological and Chemical Sciences at the University of Galway, Ireland. His primary expertise and interests are in the fields of mechanisms of cell cycle control, DNA replication and DNA damage signalling. More specifically, he is interested in the initiation of DNA replication, the role of posttranslational modifications protein–DNA and protein–protein interactions in the regulation of DNA replication and DNA repair. In recent years, he has also evolved an interest in Systems Biology. His group has established microscope techniques such as fluorescence correlation spectroscopy for Systems Biology, and he has been a collaborator in the Systems Biology Ireland network. Heinz‐Peter was previously a *FEBS Open Bio* Editorial Advisory Board Member and part of the Peer Review Task Force.

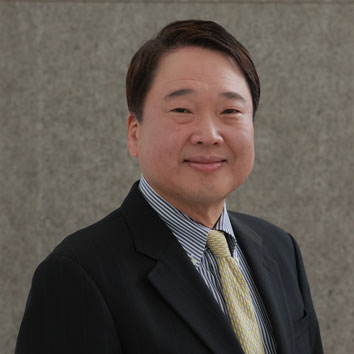

Ryuichi Morishita completed his PhD at Osaka University and he is now a professor and the Chairman of the Division of Clinical Gene Therapy at the Graduate School of Medicine, Osaka University Medical School, Japan. His research focusses mainly on gene therapy, cardiovascular disease, vascular biology, angiogenesis and dementia. In addition to his academic role, he is also a Board Chairman of the Japanese Society of Gene & Cell Therapy and a Special Advisor of Osaka Prefecture and Osaka City.

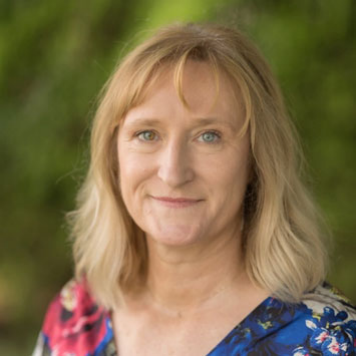

Zrinka Kovarik is permanent research adviser at the Institute for Medical Research and Occupational Medicine, Zagreb, and an associate professor of biochemistry and medicinal chemistry at the University of Zagreb, Faculty of Science, Croatia. Her research is focussed on cholinergic mechanisms and modulation of neurotransmission in poisonings and on cholinesterases—enzymes with key roles in organophosphorus poisoning and in the treatment of neurodegenerative diseases. Since March 2024, she is a member of the Executive Board, Croatian Science Foundation.

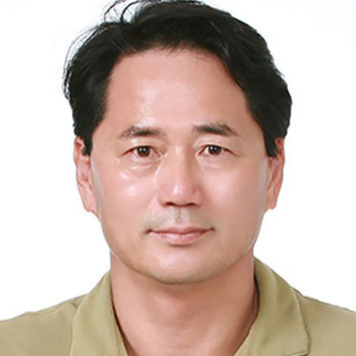

Deok Ryong Kim obtained his PhD in Biochemistry from the University of Colorado, Health Sciences Center. He is currently a professor in the Department of Biochemistry and Convergence Medical Sciences at Gyeongsang National University in Jinju, South Korea. His research primarily focusses on cancer therapy, with a particular interest in developing databases for drug repurposing and exploring the intricate role of autophagy in cancer progression and metabolic disorders.

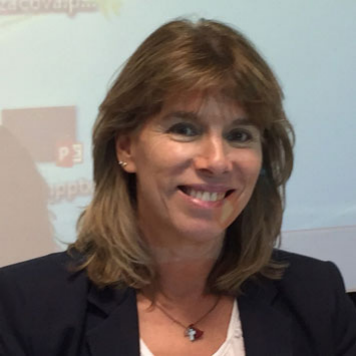

Graça Soveral obtained her PhD in Pharmacy, subarea Biochemistry, from the University of Lisbon. She is currently a full professor at Faculdade de Farmácia, Universidade de Lisboa, and principal investigator at the Institute for Research in Medicines (iMed.ULisboa) where she coordinates the Membrane Transporters in Health and Disease Group. Her main research area is membrane transport, with special emphasis on water and solute transport in cell physiology and osmoregulation, aquaporin gating and discovery of aquaporin modulators for diagnostic and therapeutic applications.

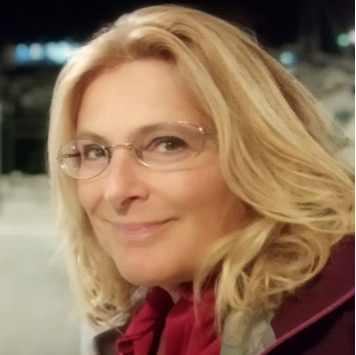

Paola Chiarugi is a full professor of biochemistry at the Faculty of Medicine and Surgery in the University of Florence and member of the Excellence at the Research Center for Transfer and High Formation. Her research is focussed on (i) the structure–function relationship of tyrosine phosphatases, (ii) the redox regulation of oxidant‐sensitive proteins during cell proliferation and cell adhesion to extracellular matrix and (ii) deregulation of tumour metabolism and energetics, as well as on cancer microenvironment. Since 2023, she is also the President of Italian Society of Biochemistry and Molecular Biology.

We were also delighted to welcome 14 new members of the Editorial Advisory Board (EAB) to the journal in 2024. The EAB is committed to helping us ensure a fast, fair and robust peer review, and we warmly welcome them to the board:

**Sangeeta Nath**

Manipal Institute of Regenerative Medicine, Manipal Academy of Higher Education (MAHE), Bangalore, India

**Sergej Pirkmajer**

Institute of Pathophysiology, Faculty of Medicine, University of Ljubljana, Slovenia

**Silvia Vega‐Rubin‐de‐Celis**

Institut für Zellbiologie (Tumorforschung), Universitätsklinikum Essen, Essen, Germany

**Sree Rama Chaitanya Sridhara**

Centro de Biología Molecular Severo Ochoa, Universidad Autónoma de Madrid, Madrid, Spain

**Pierre Santucci**

CNRS Aix‐Marseille Université, Marseille, France

**Maria E. Solesio**

Department of Biology, College of Arts and Sciences, Rutgers University, Camden, NJ, USA

**Fun Man Fung**

University College Dublin, Ireland

**Alessio Branchini**

Section of Biochemistry and Molecular Biology, Department of Life Sciences and Biotechnology, University of Ferrara, Ferrara, Italy

**Dani Osman**

Infectious Processes in Tropical Island Environments Research Unit (PIMIT), Faculty of Health, University of La Reunion Island, France

**Ioannis Kanakis**

Chester Medical School, University of Chester, UK

**Nathalie Cella**

Instituto de Ciências Biomédicas, Universidade de São Paulo, Brazil

**Sven E. Niklander**

Universidad Andres Bello, Viña del Mar, Chile

**Osamu Shimozato**

Chiba Cancer Center Research Institute, Chiba, Japan

**Erick de la Cruz‐Hernandez**

Laboratorio de Investigacion en Enfermedades Metabolicas e Infecciosas, Division Academica Multidisciplinaria de Comalcalco, Universidad Juarez Autonoma de Tabasco, Mexico

In 2024, we also saw the departure of three esteemed members of the Editorial Board: Edurne Berra, Alicia Kowaltowski and Jörg Kobarg. We greatly appreciate their support to the journal and wish them all the best in their future endeavours. Last year also brought the sad news of the passing of one of our editorial board founding members, Stuart Ferguson, who will be profoundly missed [15].

## Changes in the editorial office

After nearly 5 years as Managing Editor of *FEBS Open bio*, Duncan Wright has transitioned to the role of Managing Editor at *FEBS Letters*. During his tenure, Duncan expertly managed the day‐to‐day running of the journal and spearheaded many successful initiatives, including the *FEBS Open Bio* webinars, ‘An open chat with…’ interview series, and establishment of the Editorial Advisory Board and Peer Review Taskforce. He also played a key role in coordinating several Special ‘In the Limelight’ Issues, notably the journal's special 10th anniversary issue. We would like to thank Duncan for his exceptional contributions and wish him continued success in his new role.

Following Duncan's departure, Sara Fuentes Perez joined *FEBS Open Bio* in February 2024 as the new Managing Editor. Sara completed her PhD at University of East Anglia and John Innes Centre (Norwich), followed by postdoctoral work at University College London, working on *Drosophila* sexual antagonism. Sara started her editorial career in 2015 at *PLOS ONE* where she progressed from Associate Editor to Senior Editor, Team Manager. Before moving to *FEBS Open Bio*, she also worked for several other publishers such as Karger, Frontiers and the BMJ Group. Sara brings to the team a breadth of knowledge and experience in Open Access publishing, and we are happy to welcome her on board.

We would also like to warmly welcome Sameeha Ali, who joined the journal as our new Editorial Assistant following the full‐time transition of Irene Alverez Domenech to *Molecular Oncology*. We look forward to working with Sara and Sameeha to continue advancing the journal.

## Looking ahead to 2025

While we reflect on the developments of the last 12 months, we are excited to share our plans for the year ahead. Our first planned ‘In the Limelight’ issue will focus on education, and edited by guest editors Luciane V. Mello and Ferhan Sagin. Several other ‘In the Limelight’ issues are also in the pipeline, exploring topics such as bioinformatics, research protocols and noncoding RNAs in cancer with other special issues planned for publication later in the year. We are also looking forward to celebrating the International Day of Women and Girls in Science with the publication of a cross‐journal virtual issue with content from all four FEBS Press journals.

We are also excited to announce a new role, Publishing Liaison Officer, to further tighten the links between *FEBS Open Bio* and the scientific community. The *FEBS Open Bio* Publishing Liaison Officers will bridge the gap between the journal and scientific researchers by serving as publishing advisors and working closely in collaboration with the journal team. We also plan to more actively engage with our editorial board members through the formation of an Editorial Steering Committee. The Editorial Steering Committee will enable us to better connect with the community we serve not only by enabling a more rapid response to change but also by anticipating the changes that the scientific community expects. We hope that these new initiatives will bring us closer to the community.

Finally, we would like to thank all the authors who have chosen *FEBS Open Bio* as a home for their work, as well as all our editors, reviewers, colleagues and readers who have supported the journal in 2024. We look forward to continuing working with you all!

## Conflict of interest

The authors declare no conflict of interest.

## Author contributions

SF and MAR wrote the editorial.
